# Editorial: Epigenetic Reprogramming and Cancer Development

**DOI:** 10.3389/fcell.2020.00012

**Published:** 2020-01-28

**Authors:** Geoffrey Brown, Carolina Vicente-Dueñas, Isidro Sánchez-García

**Affiliations:** ^1^Institute of Clinical Sciences, School of Biomedical Sciences, University of Birmingham, Birmingham, United Kingdom; ^2^Institute of Biomedical Research of Salamanca (IBSAL), Salamanca, Spain; ^3^Experimental Therapeutics and Translational Oncology Program, Instituto de Biología Molecular y Celular del Cáncer, CSIC/Universidad de Salamanca, Salamanca, Spain

**Keywords:** epigenetics, cancer, stem cells, oncogenes, leukemia

For many years, scientists focused on the protein products of oncogenes driving cancer by disrupting non-nuclear events, such as the signal transduction events within cells. For example, the Ras oncogene encodes a GDP/GTP-binding guanine triphosphatase that dysregulates the signal transduction for cell growth and differentiation and the p210^BCR/ABL^ protein encoded by BCR-ABL fusion gene in chronic myeloid leukemia (CML) contributes to transformation as a constitutively active tyrosine kinase in the cytoplasm. Events that disrupt the signals cells receive from their environment are clearly important because normal cells are “socially” responsive, whereas we view the behavior of cancer cells as anarchic. Conrad H. Waddington's epigenetic landscape brought to attention the importance of a cell's “social” environment and that the signals received regulate gene expression and cell status. However, and at the time, epigenetics was in its infancy, lacking an appreciation of DNA and histone modifications that control gene expression. Nowadays, epigenetic controls on gene outputs, nurtured by environmental signals, are crucial to normal cell development and have an accepted role in the initiation and progression of cancer.

For many years, scientists have studied hematopoietic stem cells (HSC) to delineate the behavior of normal tissue-specific stem cells. They assigned each of the different leukemias to their cell-of-origin by means of a precise map of the stepwise progression of HSCs, via intermediate progenitor cells (HPCs), toward an end cell type. However, recent findings have radically changed our understanding of HSCs development. Brown and Ceredig argue that commitment/affiliation to a single cell lineage can occur within HSCs, but these cells and HPCs can still divert to another pathway. Recent findings have also brought to attention the importance of environmental signals to decision-making by HSCs. Since their discovery, we have viewed hematopoietic cytokines as supportive to cell survival and proliferation but we now know that some also instruct the choice of cell lineage by HSCs and HPCs.

Most leukemias and other cancers arise from a tissue-specific stem cell and, in contrast to the versatility of HSCs, leukemia and cancer cells often belonging to one cell lineage. One explanation is that the cell-of-origin is already lineage committed, for example, childhood acute leukemia (ALL) cells express B-cell or T-cell markers indicating a B-cell or T-cell progenitor origin, respectively. However, recent evidence favors a fetal liver HSC as the cell-of-origin and Raboso-Gallego et al. put forward a different explanation for the lineage affiliation of leukemia cells seen in childhood ALL. Their view is that an oncogene “hit-and-run” event in a HSC forces cells down a lineage by priming the epigenome of the leukemia-initiating cell (LIC). Consequentially, the leukemia stem cell arising from the LIC and partially differentiated ALL cells have the same fixed identity. NOTCH1 gain-of function mutations lead to aberrant signaling in T-cell ALL and the research article by Tottone et al. shows that epigenetic events, namely active histone H3 lysine 27 (H3K27) marks, sustain the expression of aberrant Notch3. Importantly, pharmacological inhibition of H3K27 modifiers (JMJD_3_ and p300) reduced the expression of *NOTCH3, NOTCH1*, and of their target genes. As outlined by Yang and Green, there is also a strong epigenetic component to the identity of B cell lymphomas. The B cell lymphoma (BCL6) gene encodes a transcription factor that plays a role in B cell germinal centers and complex interplay between BCL6 function and epigenetic events are of critical importance to the development of B cell lymphoma. For example, BCL6 cooperates with the *EZH2* gene that encodes a H3K27 methyltransferase and binds directly to histone demethylase LSD1 that demethylates H3K4me1/2. The findings presented support targeting BCL6 and epigenetic cross talk to treat B cell lymphoma.

As to malfunctioning of the immune system, the immunodeficiencies are a large group of different diseases that increase the risk of cancer in addition to having significant morbidity and, in some cases, mortality. Martínez-Cano et al. propose that epigenetic priming is the “third axis” to the genetic and environmental causes of immunodeficiencies, including the possibility of a triggered epigenetic mechanism. In secondary immunodeficiencies, it is increasingly becoming clear that environmental agents play a key role in triggering the epigenetic alterations.

Epigenetic programming in cancer has important implications to finding new approaches to treating, including perhaps curing, cancer. The use of tyrosine kinases inhibitors to overcome the activity of BCR-ABL protein in CML has improved treatment but not cured patients. Bugler et al. review the potential of emerging epigenetic therapies to eradicate cancer stem cells and residual disease and, in combination with current therapies, provide a cure. Epigenetic modifiers, such as SIRT_1_, HDAC, and EZH_2_ inhibitors, have been very effective in mouse models of cancer and there are in clinical trials in some cancers. The good safety profiles of modifiers in healthy individuals support trails in CML. The research report by Rivas et al. examines hepatoblastoma that is characterized by hypermethylation at particular CpG islands. As to a role for epigenetic dysregulation, they show a general disruption to the expression of genes that relate to DNA methylation, particular upregulation of *UHRF1, TET1*, and *TET2*. Furthermore, an association between a decrease in the content of 5-hydroxymethylcytosine and a poor survival rate highlights the pivotal role of epigenetics.

It seems that there is very much a call for more targeted therapies regarding the treatment of very many cancers. Epigenetic changes and reprogramming of the epigenome are both marks/events that we can readily reverse. For example and as above in the case of T-ALL, erasure of the epigenetic marks abrogated NOTCH1-driven abnormal gene expression. From this and the other scientific and clinical findings presented in this special issue, it seems that epigenomics has a bright future regarding developing a better understanding of cancer and controlling this disease.

## Author Contributions

GB wrote a draft of the Editorial that was revised by the other authors.

### Conflict of Interest

The authors declare that the research was conducted in the absence of any commercial or financial relationships that could be construed as a potential conflict of interest.

